# Printed Platinum Nanoparticle Thin-Film Structures for Use in Biology and Catalysis: Synthesis, Printing, and Application Demonstration

**DOI:** 10.1021/acsomega.2c04687

**Published:** 2023-01-04

**Authors:** Annelies Sels, Vivek Subramanian

**Affiliations:** Institute of Electrical and Micro Engineering, École Polytechnique Fédérale de Lausanne, 1015 Lausanne, Switzerland

## Abstract

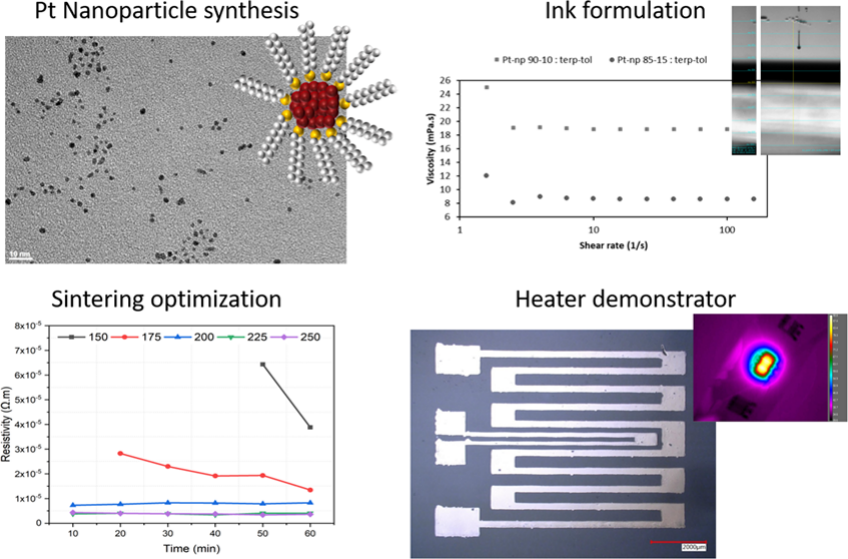

This work describes the formulation of a stable platinum nanoparticle-based ink for drop-on-demand inkjet printing and fabrication of metallic platinum thin films. A highly conductive functional nanoink was formulated based on dodecanethiol platinum nanoparticles (3–5 nm) dispersed in a toluene–terpineol mixture with a loading of 15 wt %, compatible with inkjet printing. The reduced sintering temperatures (200 °C) make them interesting for integration in devices using flexible substrates and substrates that cannot tolerate high-temperature exposures. A resistive platinum heater was successfully printed as a demonstrator for integration of the platinum ink. The platinum nanoink developed herein will be, therefore, attractive for a range of applications in biology, chemistry, and printed electronics.

## 1 Introduction

Printed metallic microstructures have been widely adopted for use in a range of applications, including printed electronics,^1−3^ photovoltaics,^4−6^ and MEMS.^7,8^ The majority of these structures have been printed using silver, due to its high conductivity and stability.^9,10^ Gold has also been used in some applications for similar reasons.^11^ For applications in biology, chemistry, and catalysis,^12,13^ there is particular interest in platinum due to its low resistance, chemical and thermal stability, catalytic activity, and biocompatibility.^14−18^

The most conventional method for homogeneous deposition of platinum thin films is physical vapor deposition (PVD).^19,20^ However, the process of creating platinum thin-film structures is complicated and expensive due to the costs and complexity associated with patterning and etching platinum using lithography, lift-off, etching, etc.

Fabrication of thin-film printed metallic patterns is fairly straightforward by droplet-on-demand inkjet printing. Inkjet printing has been widely used to structure metallic electrodes using either nanoparticle-based or organometallic inks.^21−28^

While silver and gold have received significant attention in the optimization and integration process, very little literature is reported on the use of platinum in inkjet printing.^29−32^ Although the resolution of inkjet-printed patterns is typically not as high as those formed by lithography, printing is inexpensive, effective, and versatile to make submillimeter structures distributed over large areas.

Metal nanoparticles, in comparison to their bulk, are known to have a significantly lower melting temperature. As a result, the printing and sintering processes are compatible with a broad range of substrates, including low-cost substrates such as PET and PEN. Therefore, the development of a nanoparticulate route for printing platinum is particularly attractive.

Several studies have been reported on printing aqueous platinum ink (Fraunhofer Pt-LT-20) or large particle-sized Pt nanoparticles (40 nm).^33−36^ However, inkjet printing of metallic nanoparticles suffers from nozzle clogging when using large nanoparticles, and typically necessitates the use of higher thermal budgets during sintering.^37,38^ inkjet printing of ligand-protected nanoparticles is promising as a path to addressing these shortcomings.

Stabilizing agents such as polyols and PVP are known to stabilize nanosized platinum particles.^21^ In the field of printed electronics, thiolate-protected gold and silver nanoparticles have been well studied. Thiolate-protected platinum particles have received attention in the field of catalysis; however, less so in applications involving printing. Due to their stability and unique properties, these particles are attractive for the formulation of stable printable inks. Since the particles are stabilized by encapsulants, the organic shell must be selectively removed during sintering. Any residual organic material can increase the electrical resistance of the printed film. During the sintering of the nanoparticles, the ligand–metal bonds break, resulting in the diffusion of both the ligand and the metal nanoparticles. The removal of the organic material will result in the compaction of the printed layer. The nanoparticles coalesce (necks) to form a continuous, compact, and conductive film.^39,40^ While much of this work has been performed on silver and gold, no corresponding work has been performed on platinum, which is a serious shortcoming given the attraction of platinum for use in chemistry, biology, and catalysis, all of which can benefit for printed platinum structures for use in microreactors and assay templates.

Here, we report the formulation of a stable platinum nanoparticle-based ink for drop-on-demand inkjet printing. We describe the synthesis of platinum nanoparticles with low sintering temperatures, which makes them interesting for integration in devices using low-temperature curing substrates. The novelty of this work is combining platinum nanoparticles, encapsulated by small molecules, that allow printing and sintering compatibility with a broad range of substrates. We describe their successful inkjet printing and show their use in one demonstration application as microheaters. Given the significant interest in platinum for applications in biology, chemistry, and catalysis, the realization of a robust process for additive fabrication of platinum thin-film structures is therefore particularly attractive in this regard.

## 2 Experimental Section

### 2.1 Nanoparticle Synthesis

As a first step toward realizing a viable ink for the printing of platinum, we evaluated two different classes of platinum nanoparticles with different ligands. Thiolate-protected platinum nanoparticles were synthesized via two bottom-up methods, based on previous methods described in the literature. Castro et al.^41^ based their synthesis process on a modified Brust method using thiols as stabilizing ligands (hexanethiol and dodecanethiol), while San et al.^42^ used a noncommercial sodium S-octylthiosulfate ligand as a stabilizing agent.

Castro et al.:^41^ An aqueous solution (50 mL) of the metal ion precursor hydrogen hexachloroplatinate(IV) hydrate (H_2_PtCl_6_·*X* H_2_O, 1 g) was added to a solution of 4.22 g of tetraoctylammonium bromide (TOABr) in 150 mL of toluene and stirred for 15 min. Then, an aqueous solution of 0.803 g of sodium borohydride (NaBH_4_) in 50 mL of H_2_O was added quickly to the mixture. Ninety seconds after addition, 0.925 mL of the dodecanethiol ligand was added to the solution and stirred for 3 h at room temperature. The yellow suspension turned into a dark solution, indicating the further reduction of Pt(I) to Pt(0) and therefore formation of nanoparticles. After 3 h, purification of the reaction mixture started by removing the aqueous phase and evaporation of the organic phase. Methanol was added to the dried product to remove free thiol and other byproducts. This solution was centrifugated for 3 min at 9000 rpm and the precipitate was redissolved in dichloromethane (DCM). This was repeated two times. Next, the product was dissolved in DCM and passed through a PTFE syringe filter (0.2 μm) to remove insoluble byproducts.

San et al.:^42^ 6 mL of 1-bromododecane was mixed with 50 mL of ethanol, and 6.2 g of Na_2_S_2_O_3_·5H_2_O was dissolved in 50 mL of water. Both solutions were mixed in a 250 mL round-bottom flask, which was then connected to a reflux condenser. After the solution mixture was refluxed for 3 h, the resulting ethanol was removed by a rotary evaporator. The final solution was cooled to room temperature. The white solid product was isolated, dissolved in hot ethanol, and recrystallized to form a crystalline solid. 1H NMR confirmed the formation of the ligand (see the Supporting Information).

Hydrogen hexachloroplatinate(IV) hydrate (H_2_PtCl_6_; 0.4 mmol) was dissolved in 12 mL of nanopure water, and TOABr (2.0 mmol) was dissolved in 25 mL of toluene. Two solutions were mixed and stirred for ∼15 min. After the phase transfer, the aqueous layer was separated and discarded by a separatory funnel. The synthesized sodium S-dodecylthiosulfate ligand (0.8 mmol) was dissolved in 10 mL of 25% methanol. The ligand and TOABr (2.0 mmol) were added to the separated organic layer, and the reaction mixture was stirred for 15 min. NaBH_4_ (8.0 mmol) was dissolved in 7 mL of nanopure water before it was added to the vigorously stirring reaction flask within 10 s. The reaction mixture first turned dark orange and then black, which indicated the formation of Pt nanoparticles. The reaction was stirred for an additional 3 h and the washing step was similar to the first method.

Inks were formulated for inkjet printing based on the synthesized platinum nanoparticles. A nanoparticle loading of 15 wt % was chosen. A solvent mixture of 10 wt % toluene and 90 wt % α-terpineol matched the required viscosity range (8.5 mPa·s) and was able to disperse the nanoparticles well. Filtration through a PTFE syringe filter (0.2 μm) was required to remove any insoluble byproducts and avoid clogging the printhead.

### 2.2 Characterization

TEM images were taken using an FEI Tecnai Osiris instrument at an accelerating voltage of 120 kV. The samples were prepared by drop-casting 2 μL of the nanoparticles in dichloromethane onto a carbon-coated copper 400 mesh grid, followed by drying under ambient conditions.

A Discovery HR-2 rheometer (TA Instruments) was used to determine the viscosity of the inks at 25 °C, with varying shear rates between 1 and 200 1/s. All pure solvent systems were considered to be Newtonian. Addition of nanoparticles can increase an ink’s low shear rate viscosity and lead to non-Newtonian behavior at sufficiently high particle loading (∼60%).^43,44^ However, non-Newtonian behavior was not observed during jetting (at shear rates of ∼10^5^ 1/s), and thus, viscosity values measured at low shear rates were used. TGA was performed on a Linseis TGA PT1600, and SEM analysis was recorded on a Gemini SEM 450 (Zeiss) at an accelerating voltage of 5 kV.

The printing optimization was performed using a Dimatix Materials Printer (DMP-2850, Dimatix-Fujifilm). The nominal ejection volume of the printhead (DMCLCP-11610, Dimatix-Fujifilm) was 10 pL. The nozzle for ink ejection was controlled by a bipolar waveform, the nozzle was heated up to 30 °C, and the printing stage was set at 58 °C. Jetting was set at a frequency of ∼20 Hz and ejected at 6 m/s.

Using the aforementioned inks and printing processes, demonstration microheaters were printed on glass substrates. Each microheater also included an integrated resistive temperature detector (RTD). Calibration of the heater was performed by probing two probe tips on each printed pad of the heater while ramping up the temperature of the measurement stage to a set value. The sensing measurements were performed by placing two probe tips on the printed pads of the heater and two probe tips on the printed pads of the RTD to allow for simultaneous heating and temperature measurement. A Keithley 4200A-SCS was used to record all measurements.

## 3 Results and Discussion

**Figure 1:**
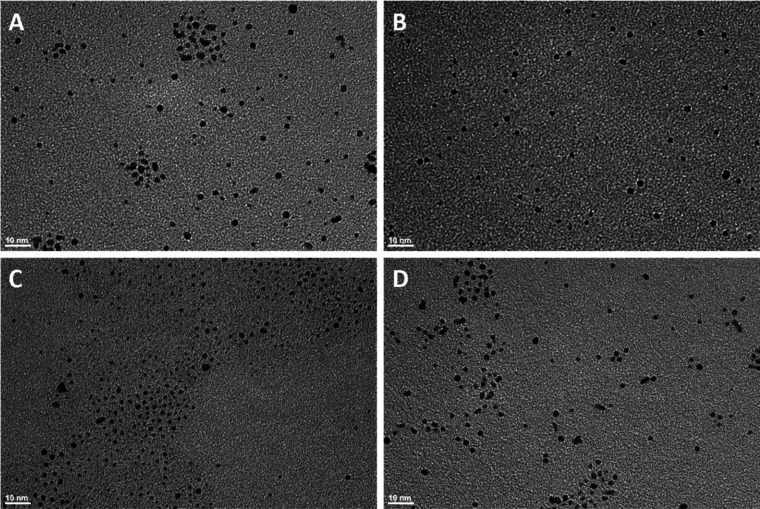
TEM of platinum nanoparticles with hexanethiol (A; 2.4 ± 0.5 nm), octanethiol (B; 2.5 ± 0.4 nm), dodecanthiol (C; 2.3 ± 0.6 nm), and dodecylthiosulfate (D; 2.2 ± 0.3 nm) ligands. The scale bar represents 10 nm.

Castro et al. based their synthesis process on a modified Brust method using thiols as stabilizing ligands: CH_3_(CH_2_)*_n_*SH/hexanethiol (*n* = 6), octanethiol (*n* = 8), and dodecanethiol (*n* = 12). NaBH_4_ is not able to completely reduce the Pt–S species formed after addition of the ligand; it was therefore suggested to reverse the order of addition. Although this method results in the successful formation of platinum nanoparticles, we observed that the majority of the platinum salt was unable to react and produce nanoparticles, resulting in a very low yield after synthesis. TEM confirmed the formulation of platinum particles of 2–4 nm (Figure 1a–c).

San et al.^42^ developed a different synthesis procedure using the noncommercial sodium S-alkylthiosulfate ligand as a stabilizing agent. Addition of this thiosulfate ligand to the platinum precursor resulted in the formation of Pt(SR)_2_ complexes. Reduction of the Pt–S species by NaBH_4_ resulted in stable thiolate-protected platinum nanoparticles with an increased yield. During synthesis, a gradual color change of the initially light yellow Pt(IV) solution to brown (representing Pt(II)) and finally from brown to black (marking the formation of Pt(0)) was observed. The mechanism was followed by NMR^45,46^ and confirmed the chemisorption of ligands as thiolate on the Pt surface, forming a monolayer. Therefore, this latter method was chosen as preferential. TEM (Figure 1c,d) confirmed the formulation of identical dodecanthiol platinum nanoparticles using both methods.

**Figure 2:**
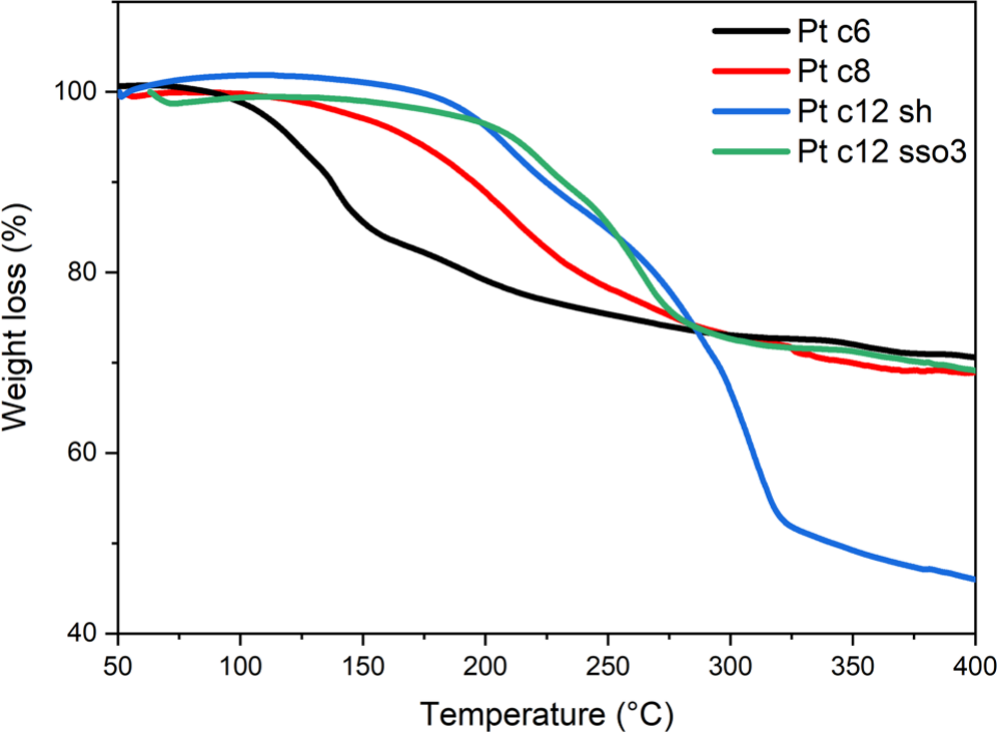
TGA analysis of platinum nanoparticles with hexanethiol (Pt 6), octanethiol (Pt c8), dodecanthiol (Pt c12 sh), and dodecylthiosulfate (Pt c12 sso_3_) ligands.

Additional characterization by TGA (Figure 2) elucidates the sintering behavior of the nanoparticles and shows mass loss through ligand removal as a function of temperature.

TGA analysis demonstrates the increased sintering temperature required for larger ligands. The onset conversion temperature increases from ∼120 °C for hexanethiol particles to 200 °C for dodecanethiol particles. This observation agrees with previous sintering studies on gold nanoparticles. An increased weight loss can be observed for dodecanethiol compared to dodecylthiosulfate-synthesized particles resulting from incomplete removal of the free ligands.

### 3.1 Printing and Sintering Optimization

Formulation of nanoparticle inks for inkjet printing is a well-studied field.^1,3,11,25^ The choice of a binary solvent system for this ink formulation is opted to prevent the commonly observed coffee-ring effect during drying. For this ink, α-terpineol and toluene were chosen. Toluene is a known solvent for the dispersing of the nanoparticles, while terpineol is used to modify the viscosity of the ink to match the viscosity requirements for inkjet printing (5–12 cP).

**Figure 3:**
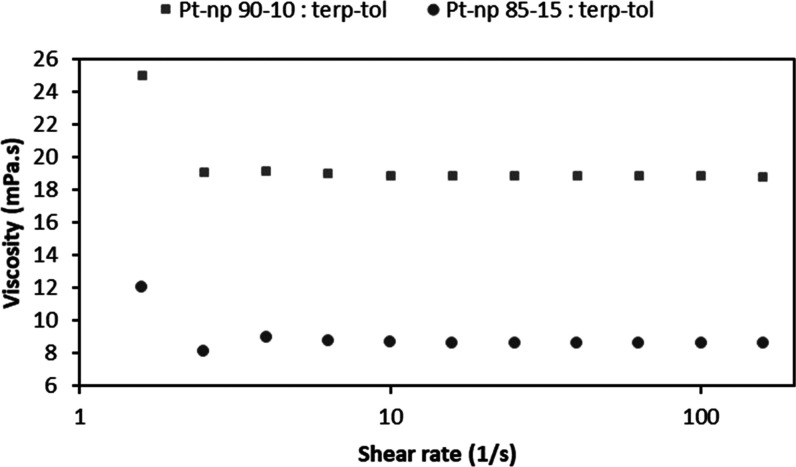
Rheometry studies of platinum nanoparticles in 10 and 15 wt % toluene.

Two inks were formulated containing toluene and terpineol in toluene/terpineol ratios of 10:90 and 15:85 wt %. Both inks were formulated with a nanoparticle loading of 15 wt %, which was found to deliver stable inks with the appropriate viscosity for inkjet printing. The rheometry analysis shown in Figure 3 compares these two ink compositions. A clear Newtonian behavior was observed, with ink viscosities of 19 and 8.5 mPa·s, respectively. The latter composition (10: 90 wt %) was therefore found to be ideal for inkjet printing.

The viscosity of the formulated ink is compatible with inkjet printing using a Dimatix DMP-2850 printer. The printing parameters were optimized to achieve stable drop formation. The applied waveform is a standard bipolar pulse waveform, producing drops with calculated volumes of ≈10 pL. The jetting frequency was set to 2 kHz, the nozzle firing voltage was set to 35 V, and the nozzle temperature was set to 30 °C. The printing stage was set at 58 °C and was allowed to settle before printing began to avoid temperature fluctuations during printing.

**Figure 4:**
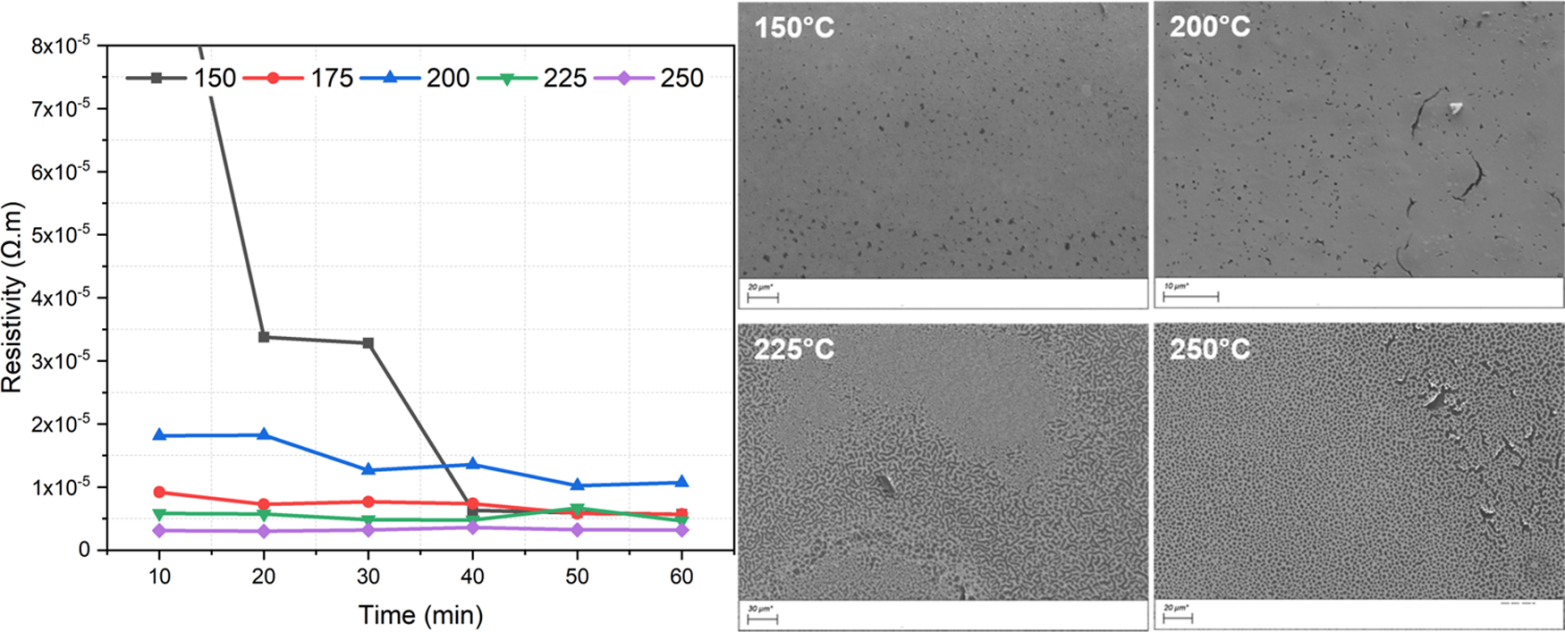
Sintering studies of hexanethiol, 3.2 × 10^–6^ Ω·m, and 0.055 μm thick. The scale bar represents 20 μm (150 °C), 10 μm (200 °C), 30 μm (225 °C), and 20 μm (250 °C).

**Figure 5:**
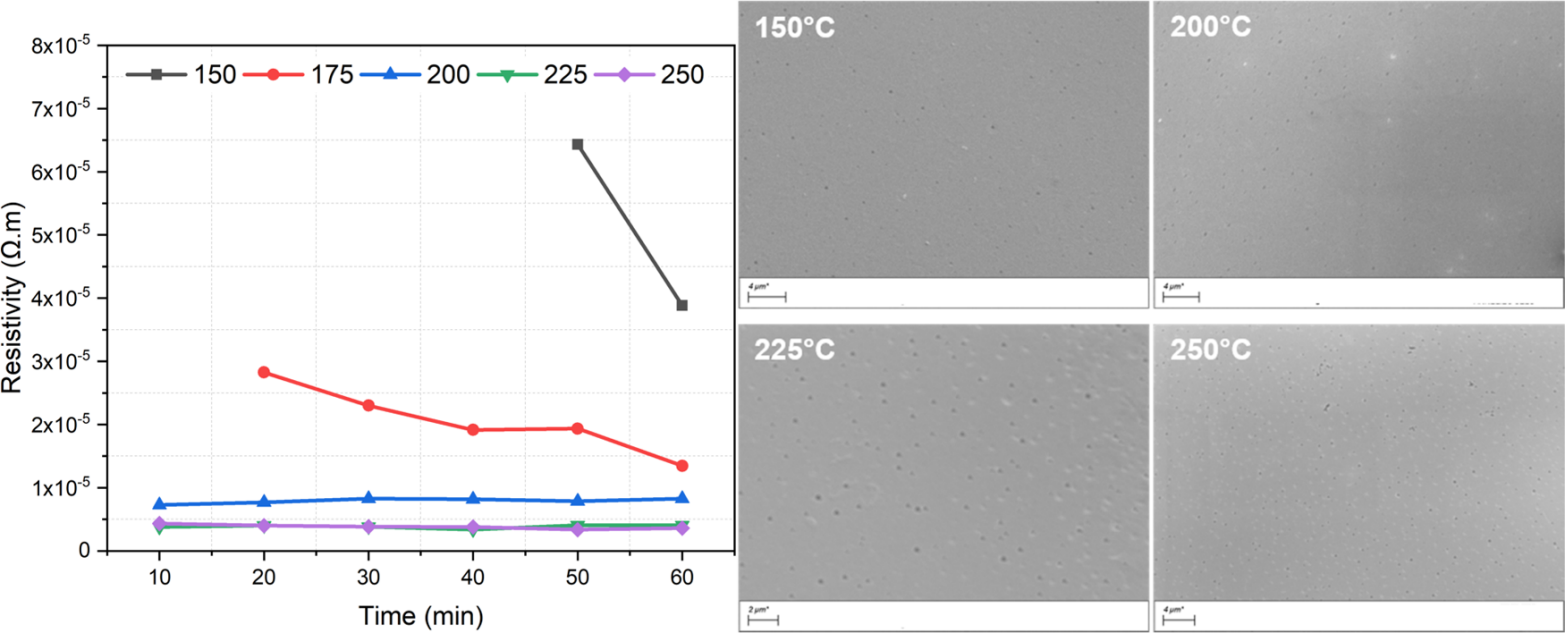
Sintering studies of dodecanethiol, 3.6 × 10^–6^ Ω·m, and 0.02 μm thick. The scale bar represents 4 μm (150 °C), 4 μm (200 °C), 2 μm (225 °C), and 4 μm (250 °C).

The aforementioned optimized printing conditions of the ink were used to perform a sintering study. Printed squares were sintered at different temperatures, following the resistance of the film as a function of time. Their corresponding SEM images are shown in Figures 4 and 5.

Printed films of hexanethiol-protected platinum nanoparticles (*n* = 6) were sintered at 150, 175, 200, 225, and 250 °C for 60 min. The resistivity was measured at intervals of 10 min at the indicated temperatures. For each resistivity measurement, 5 replicates were measured. The films exposed to the lowest curing temperature (150 °C) required 20 min to convert into a partially conductive layer (3.5 × 10^–5^ Ω·m). Increasing the sintering time at 150 °C to 40 min resulted in conductive films (1 × 10^–5^ Ω·m). A similar resistivity was observed at higher temperatures, from 175 °C, after only 10 min of curing time. SEM confirms the formation of a homogeneous layer of platinum. Note that the 200 °C samples show slightly higher resistance, likely due to some cracking evident in these samples.

A similar sintering study was conducted on dodecanethiol-protected platinum nanoparticles (*n* = 12, Figure 5). Due to the higher carbon content in this ink, the printed features require a higher sintering temperature. Curing at 150 °C for 60 min is not able to completely convert the dodecanethiol films. Increasing the curing temperature to 175 °C improves conversion; however, 60 min of curing cannot produce a completely cured platinum layer. At temperatures above 200 °C, curing for short periods is sufficient to produce conductive printed layers (1 × 10^–5^ Ω·m). These sintering conditions make the platinum nanoparticle ink compatible with numerous flexible substrates.

**Figure 6:**
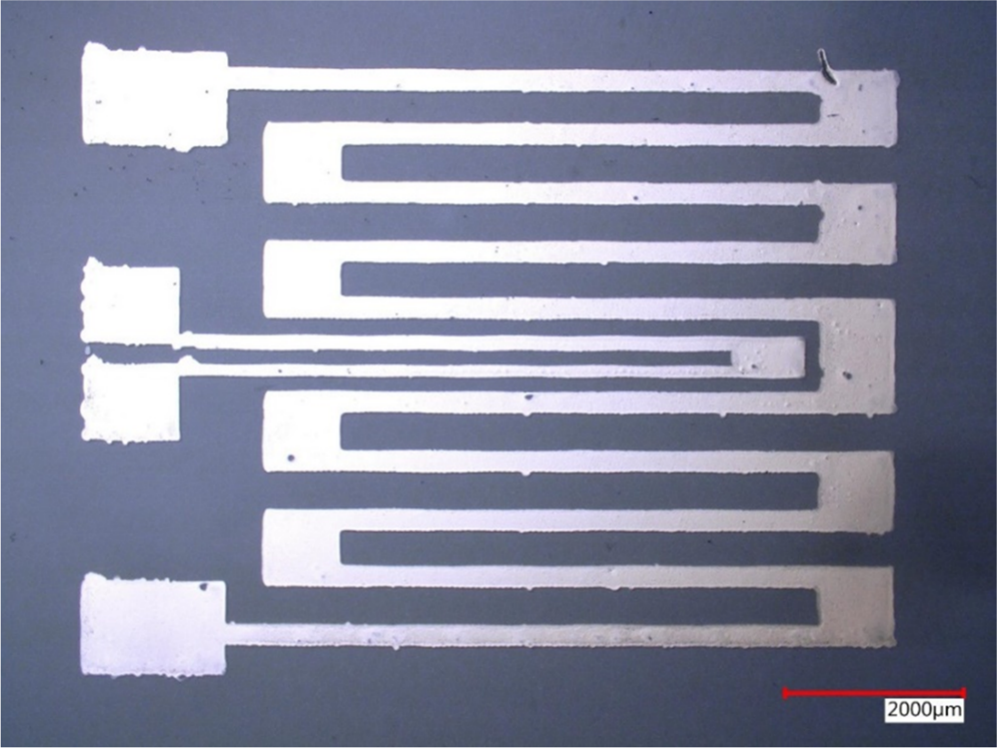
Resistive heater with an integrated temperature sensor. Sensing measurements were performed by probing two probe tips on the printed pads of the heater (major serpentine) and two probe tips on the printed pads of the RTD (middle feature).

As a demonstrator for integration of platinum ink, a resistive platinum heater was printed. Platinum heaters were printed on glass substrates for convenience; hence applications such as disposable PCR modules can benefit from integration of these heaters. Figure 6 shows the printed serpentine heater pattern with an integrated temperature sensor.

Since nanoparticle inks contain high quantities of the organic material from the stabilizing ligands, significant compaction (70–80%) is observed during sintering. Deposition of four consecutive printed layers results in a sintered thickness of 0.3 μm. To reduce cracking during this specific sintering process, sintering was performed every two layers at 200 °C.

The Pt heater was fabricated as follows: First, the glass substrates were cleaned thoroughly with IPA and acetone during sonication. Next, the substrates were transferred to a Dimatix DMP-2850 printer with the printing plate temperature set at 58 °C. The structures were printed with 5 min drying time between the layers. After every two layers, the substrates were transferred to a hotplate for curing. Figure 6 illustrates the fabricated heater integrated with an RTD. The resistances of the Pt heater and Pt RTD at a room temperature of 20 °C were 300 and 670 Ω, respectively.

### 3.2 Application: Printed Resistive Heater

Resistive platinum heaters are a good candidate to demonstrate the usefulness of the ink. Microheaters have been extensively investigated because of their broad range of applications due to their high-temperature resistance coefficient (TRC), high sensitivity, and fast response.^47^ Platinum is preferred over gold because of the increased bulk resistance, which is preferential for RTD devices and to avoid the disadvantage of the softening of gold at higher temperatures.^48,49^

Resistive heaters are operated by flowing an electrical current through the serpentine. By applying a bias current to the resistive heater, the energy is converted into heat as it flows through the resistance through joule heating.

**Figure 7:**
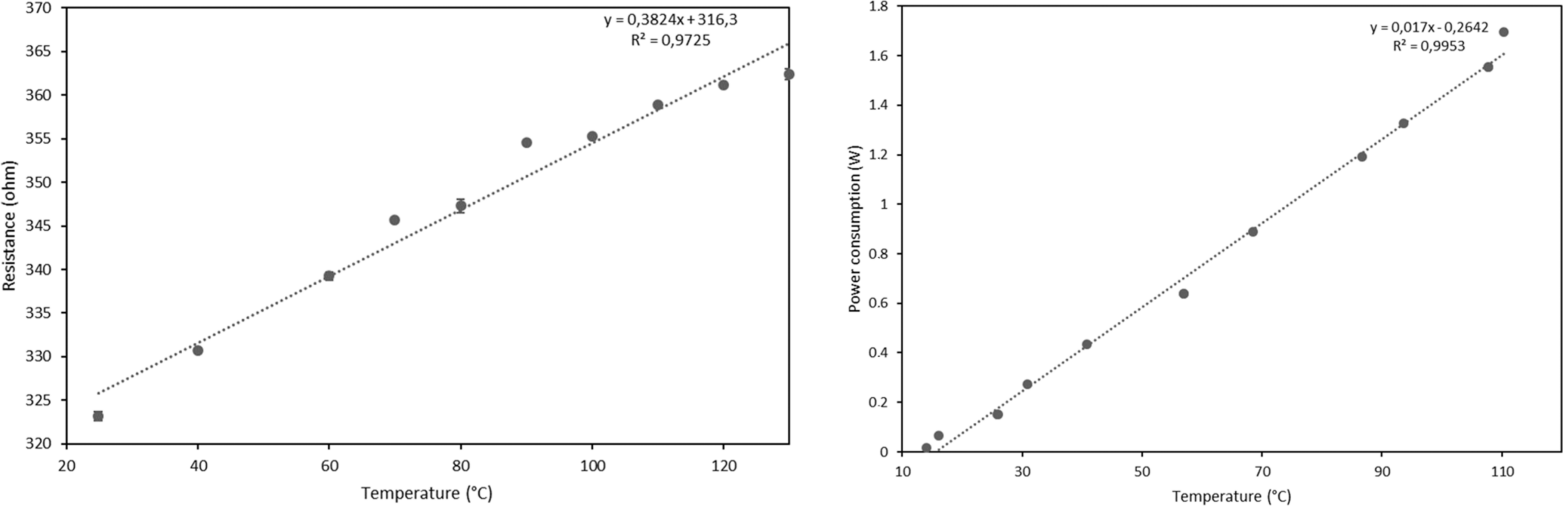
Calibration (left) and power consumption (right) of the printed heater.

Calibration of the heater was performed using a Keithley semiconductor parameter analyzer and a probe station equipped with a heated stage. The resistance of the Pt RTD sensor was measured at different temperatures between room temperature and 130 °C. The calibration curve (Figure 7) indicates a fairly linear resistance variation as a function of temperature. Five replicate cycles were performed. Additionally, the cyclability was confirmed by heating and cooling the same heater multiple times and measuring the resistance at 80 °C. The error bar shown in Figure 7 represents the variation in resistance as a function of up to five cycles, attesting to excellent cyclability.

Once the calibration of the integrated temperature sensor was performed, the function of the heater was evaluated. Figure 7 shows the effect of heating power on the achieved temperature. As expected, a linear correlation is observed between the applied power and the achieved temperature. Around 1 W is required to heat the RTD up to 80 °C. The maximum temperature limit of this resistive heater was not evaluated due to limitations of the power delivery capability of the current source. However, temperatures around 100 °C could be easily achieved.

**Figure 8:**
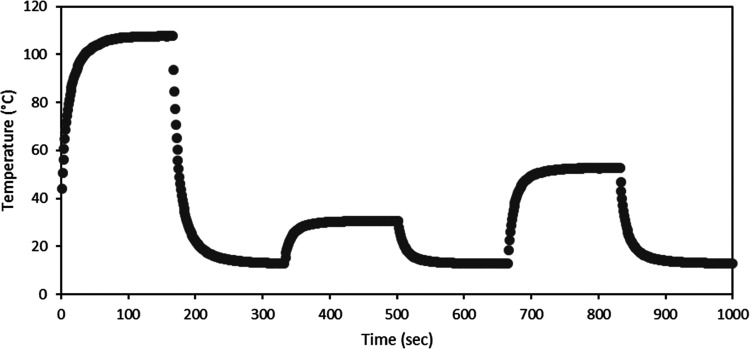
Recovery of the heater: thermal cycling evaluation of the printed resistive heater. Temperature–time curve evaluating the recovery of the heater at 45 mA (107.7 °C), 20 mA (30.8 °C), and 30 mA (57.0 °C).

Besides the temperature range and power consumption required for the heaters to function, the recovery of the heaters to room temperature is also a critical factor to consider. Thermal cycling evaluation experiments for recovery were carried out by monitoring the temperature as a function of time, as shown in Figure 8. The recovery of the heaters was studied at various temperatures (current/power levels), e.g., 107.7 °C (45 mA, 1.55 W), 30.8 °C (20 mA, 0.27 W), and 57.0 °C (30 mA, 0.63 W). The recovery times were found to be 120, 90, and 100 s, respectively. It should be noted that a sufficient long heating time (160 s) of heating is required to ensure temperature stability prior to measuring the real recovery time.

To summarize, a resistive heater with an integrated RTD sensor was successfully printed using the platinum nanoparticle ink. Good pattern fidelity and quality were achieved, in line with the expectations for inkjet printing. The functionality of the heater was evaluated and a linear correlation between power consumption and temperature could be observed. In addition, thermal recovery after heating up to 100 °C could be observed, as well as a return to the original state of the heater.

## 4 Conclusions

Thiolate ligand-stabilized platinum nanoparticles (3–5 nm) were synthesized and used to create a highly conductive functional nanoink. A stable ink using a 10–90 toluene–terpineol combination was developed to ensure the aggregation stability of nanoparticles. A nanoparticle loading of 15 wt % resulted in a stable ink (8.5 cP), compatible with inkjet printing. Using the dodecanethiol ligand has the advantage that at temperatures above 200 °C, curing for short periods is sufficient to result in conductive printed layers (1 × 10^–5^ Ω·m). These sintering conditions make the platinum nanoparticle ink compatible with numerous flexible substrates. Additionally, as a demonstrator, a resistive platinum heater was successfully printed. A linear correlation between power consumption and temperature up to 100 °C and a reasonable recovery of the heater could be observed. This attests to the potential of the platinum nanoink herein for use in various applications in printed electronics.

## Supporting Information Available

^1^H NMR spectrum of sodium S-dodecylthiosulfate ligand and additional characterization of the printed resistive heater (PDF)

## Abbreviations

MEMS: microelectromechanical systems
PVD: physical vapor deposition
PET: poly(ethylene terephthalate)
PEN: poly(ethylene naphthalate)
PVP: poly(vinylpyrrolidone)
RTD: resistance temperature detector
PCR: polymerase chain reaction
