# Supplementary material for: Printed Platinum Nanoparticle Thin-Film Structures for Use in Biology and Catalysis: Synthesis, Printing, and Application Demonstration
Source: ACS Omega. 2023 Jan 4;8(2):1929–36. doi: 10.1021/acsomega.2c04687 (PMC9850773; doi:10.1021/acsomega.2c04687)

Supporting information:

Printed platinum nanoparticle thin film structures for use in biology and catalysis: Synthesis, printing, and application demonstration

*Annelies Sels, Vivek Subramanian**

Institute of Electrical and Micro Engineering, École Polytechnique Fédérale de Lausanne, Switzerland

*Corresponding author

Figure S1: H-NMR of sodium S-dodecylthiosulfate ligand

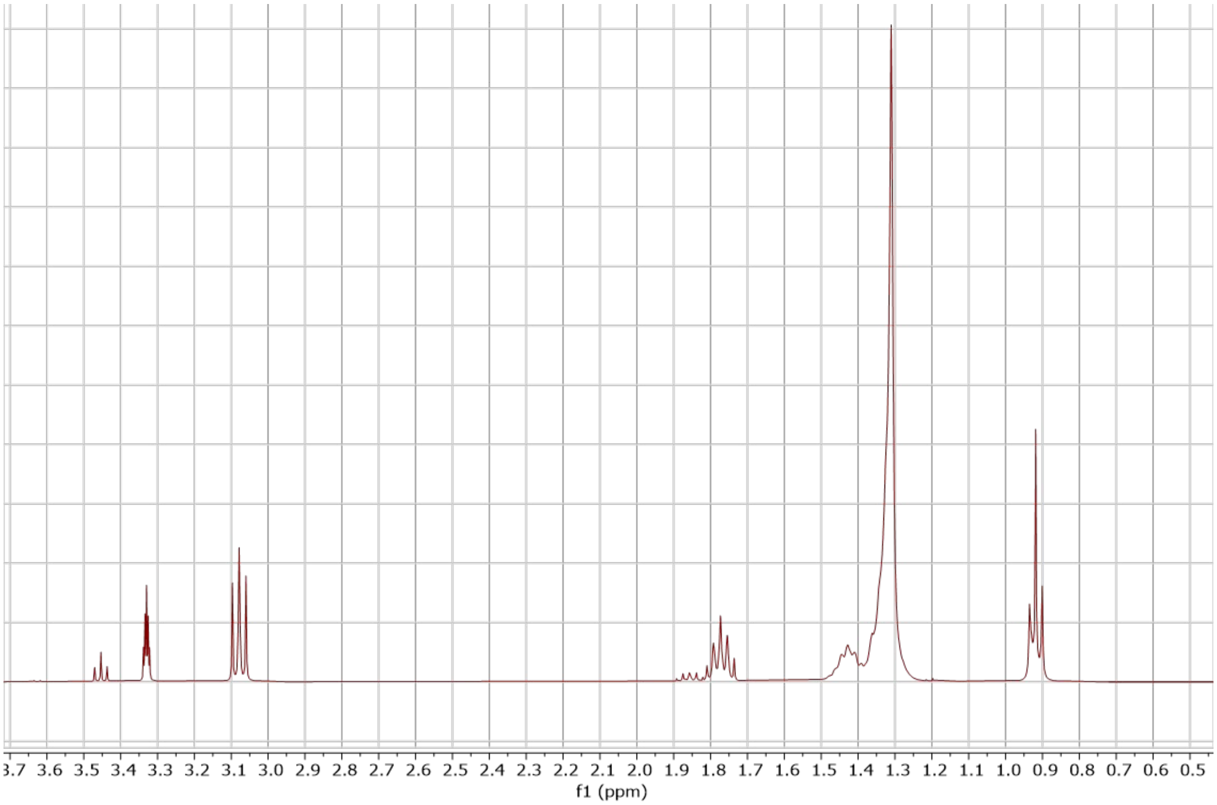

^{1}H NMR (400 MHz, D_2O): triplet (δ 3.1 ppm, α-CH_2−S), quintet (δ 1.7 ppm, β-CH_2CH_2−S), broad peak (δ 1.3 ppm, −CH_2−), and another triplet (δ 0.9 ppm, CH_3−). Impurities are from 1-Bromodecane: triplet (δ 3.45 ppm, α-CH_2−), triplet (δ 3.34 ppm, α-CH_2−Br), quintet (δ 1.85 ppm, β-CH_2CH_2−Br), broad peak (δ 1.4 ppm, −CH_2−), and another triplet (δ 0.9 ppm, CH_3−).

Figure S2: Repeatability of the heater

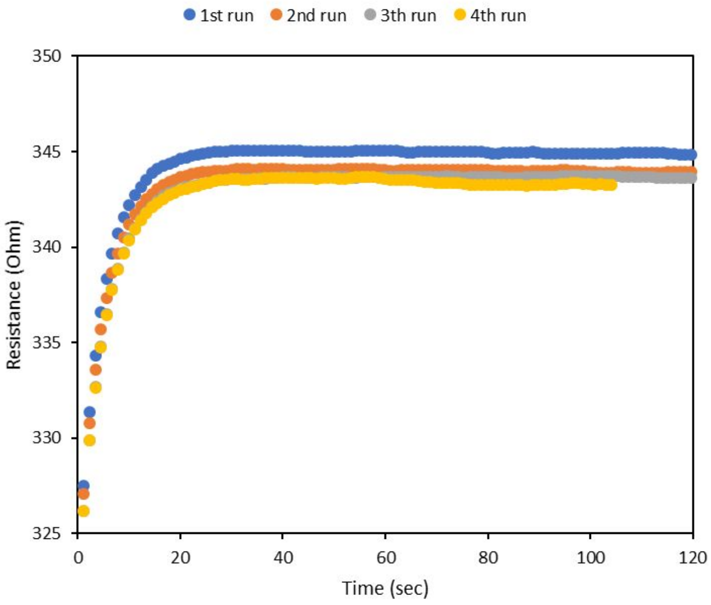

Figure S3: Uniformity

Using the IR camera, the uniformity of the heater can be observed and the temperature during functioning confirmed. The emissivity used for these heaters is 0.9.

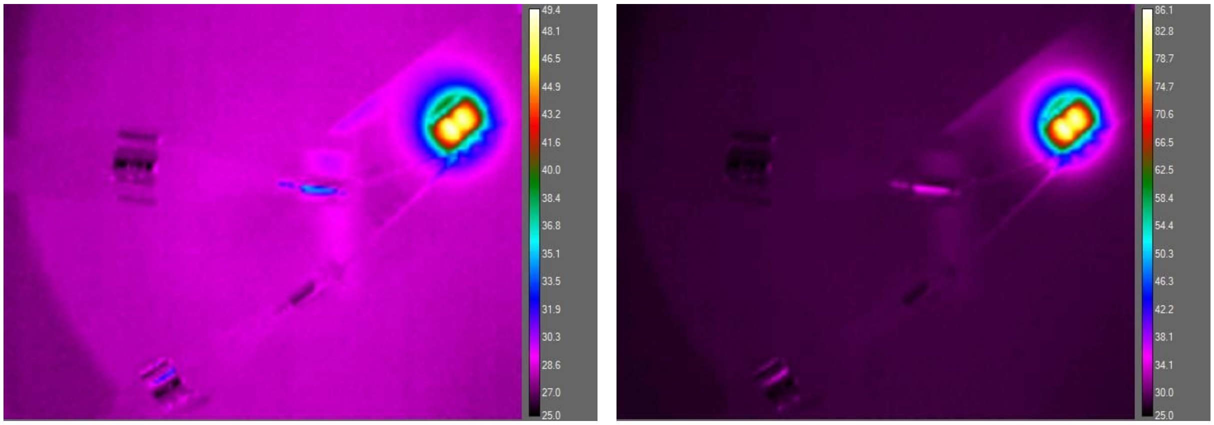

Supplement: Supplementary file 1 — ao2c04687_si_001.pdf [file ao2c04687_si_001.pdf]
